# Strategies for Signal Amplification of Thyroid Hormones via Electromigration Techniques Coupled with UV Detection and Laser-Induced Fluorescence

**DOI:** 10.3390/ijms26083708

**Published:** 2025-04-14

**Authors:** Michał Pieckowski, Ilona Olędzka, Tomasz Bączek, Piotr Kowalski

**Affiliations:** 1 Department of Pharmaceutical Chemistry, Medical University of Gdańsk, Hallera 107, 80-416 Gdańsk, Poland; mpiec@gumed.edu.pl (M.P.); ilona@gumed.edu.pl (I.O.); tbaczek@gumed.edu.pl (T.B.); 2 Department of Nursing and Medical Rescue, Institute of Health Sciences, Pomeranian University in Słupsk, 76-200 Słupsk, Poland

**Keywords:** thyroid hormones, signal amplification, pressure-assisted electrokinetic injection, micellar electrokinetic capillary chromatography, urine samples

## Abstract

Several strategies, including UV detection with a diode array detector (DAD), laser-induced fluorescence (LIF), derivatization reactions, the use of micelles in the separation buffer, as well as online preconcentration techniques based on pressure-assisted electrokinetic injection (PAEKI), and offline preconcentration using solid-phase extraction (SPE) columns containing quaternary amine groups with a chloride counterion, were investigated for the simultaneous separation and signal amplification of free thyroid hormones (THs) in biological samples. Moreover, a sensitive method for the quantification of THs in selected biological samples using micellar electrokinetic capillary chromatography with LIF detection (MEKC-LIF) was developed. The THs present in biological samples (L-tyrosine, T2, T3, rT3, T4, and DIT) were successfully separated in less than 10 min. The analytes were separated following a derivatization procedure with fluorescein isothiocyanate isomer I (FITC). A background electrolyte (BGE) composed of 20 mM sodium tetraborate (Na_2_B_4_O_7_) and 20 mM sodium dodecyl sulphate (SDS) was employed. Key validation parameters such as linearity, precision, limits of detection (LOD), and limits of quantification (LOQ) were determined. The use of PAEKI for the electrophoretic determination of free THs demonstrates significant potential for monitoring these hormones in real urine samples due to its high sensitivity and efficiency.

## 1. Introduction

Hormones produced by the thyroid gland are essential for the proper development and functioning of nearly all tissues and organs in the human body, including the heart, lungs, kidneys, liver, central nervous system, and reproductive organs. Their role is indispensable throughout all stages of human life. The concentration of thyroid hormones significantly influences many vital processes, such as fetal development; brain development and function; peripheral nervous system activity; calcium-phosphate metabolism; protein, fat, and carbohydrate metabolism; water balance; energy transformations and heat production; skeletal growth and maturation; and muscle strength regulation [1,2,3].

The thyroid gland produces hormones 3,3′,5-triiodo-L-thyronine (T3) and 3,5,3′,5′-tetraiodo-L-thyronine (T4) in response to thyroid-stimulating hormone (TSH), secreted by the pituitary gland. This gland also synthesizes hormones 3,3′,5′-triiodo-L-thyronine, reverse T3 (rT3), 3,3′-diiodo-L-thyronine (T2), 3′-monoiodo-L-thyronine (T1), and calcitonin. THs interact with various substances, including insulin, cortisol, and sex hormones. Among these, T4 is the primary hormone secreted by the thyroid gland, and T3 is converted from T4 in the kidneys and liver through deiodination of the phenolic ring [4]. Although T3 is more biologically active than T4, it is present in concentrations that are approximately one magnitude lower than the total amount of T4 in healthy individuals [5]. T3 acts as a metabolically active hormone that binds to nuclear receptors to form an active hormone-receptor complex, thereby influencing gene expression. This process enhances metabolic transformations, increases oxygen consumption, and generates heat. At physiological concentrations, T3 exhibits anabolic effects; however, at elevated levels, it can have catabolic effects that lead to protein breakdown in muscles [6].

The biosynthesis and secretion of iodothyronines are regulated by a negative feedback system involving the pituitary gland, hypothalamus, and thyroid gland. Iodine plays an invaluable role in synthesizing thyroid hormones; it is obtained from food and absorbed in the stomach and small intestine before being transported to the thyroid gland for storage in follicular cells [7]. Under the influence of thyroid peroxidase (TPO), free iodine is incorporated into the tyrosine residues of thyroglobulin to form 3-iodo-L-tyrosine (MIT) and 3,5-diiodo-L-tyrosine (DIT), which couple to produce T4 and T3 [8].

Currently, considerable attention is focused on studying the impact of THs on fetal development [9], skeletal formation [10], the occurrence of osteoporosis [11], osteoarthritis [12], the maintenance of normal neurological functions through effects on oligodendrocyte maturation and myelination [13], inflammation [14], and oxidative stress [15]. Research has demonstrated that THs significantly afect reproductive capabilities in both sexes [16].

Immunometric measurement of TSH serves as the gold standard in the diagnosis of thyroid gland dysfunctions, with the sensitivity of such tests being approximately 0.02 mIU L^−1^ [17]. However, in cases where hypothyroidism is suspected, reference ranges for TSH levels are not sufficient for diagnostic purposes, and additional measurement of free T4 is recommended [18]. Studies have reported a stronger association between disturbances in the cardiovascular, skeletal, and metabolic systems and T4 levels than with TSH [19]. Most laboratories measure free or total T4 and T3 using radioimmunological [20] or immunological [21] methods. While these methods exhibit high sensitivity, they may sometimes lack specificity for THs due to various interferences, including endogenous antibodies and the presence of abnormal binding proteins or free fatty acids. These factors can lead to falsely elevated or decreased results [18]. It is crucial to note that only unbound hormones—specifically, the free forms—are biologically active. Regarding THs, approximately 75% of T4 and T3 exist in bound form, indicating a minimal percentage of clinically available THs. Furthermore, it is important to emphasize that binding protein concentrations are influenced by numerous factors, including health status [22]. Consequently, clinicians are particularly interested in obtaining serum-free THs (the active unbound forms), which are essential for diagnosing thyroid diseases. Additionally, the measurement of rT3 allows for differentiation between hypothyroidism and low T3 syndrome, which can occur in various chronic diseases and critical conditions. Recently, there has been increased interest in the relationship between rT3 and T3 levels concerning cell proliferation and cancer cell growth [23,24].

In the field of separation sciences, liquid chromatography coupled with mass spectrometry (LC-MS) is the method of choice for the analysis of THs [1,2,25,26]. Furthermore, high-performance liquid chromatography (HPLC) combined with UV spectrophotometry [27,28], ultra-high performance liquid chromatography with mass spectrometry (UHPLC-MS) [29,30], and gas chromatography with mass spectrometry (GC-MS) [31] have also been employed in these analyses. Moreover, there are published studies utilizing electromigration techniques combined with spectrophotometric [32] and amperometric [33] detectors, as well as mass spectrometry [34]; however, these methods often lack sufficient sensitivity or focus on the analysis of individual analytes. Table 1 summarizes the latest methods for THs determination.

The primary objective of this study was to present various signal enhancement strategies for THs while simultaneously quantifying L-Tyr, T2, T3, rT3, T4, and DIT. Various approaches were employed, including DAD and LIF, optimization of THs derivatization using fFITC, and the application of micelles in the separation buffer. Water-wettable EVOLUTE EXPRESS AX SPE columns containing quaternary amine groups with a chloride counterion were first used for the isolation of THs from biological samples. Additionally, for the first time, online preconcentration techniques based on -PAEKI- was combined with a LIF detection. To the best of our knowledge, this is the first attempt to compare these methodologies for the determination of THs. Furthermore, it is the first study that describes the simultaneous quantification of six FITC-THs derivatives using electromigration techniques coupled with a LIF detector.

## 2. Results and Discussions

### 2.1. Optimization of the Derivatization Procedures

In the selection of the THs derivatization agent, the following fluorescent derivatives were tested: carboxyfluorescein succinimidyl ester (CFSE), 5-diethylaminofluorescein (5-DTAF), and FITC. These procedures for the derivatized compounds were based on the publication by Molina and Silva [35], where the pH range of the reaction mixture should be in the range of pH 9.5–9.6. To standard solution samples containing a mixture of the analytes at a concentration of 10 µg mL^−1^, the derivatization buffer and labeling solution were added in Eppendorf tubes to obtain a final volume of 100 µL. The details of the derivatization procedures for the tested compounds are collected in Table 2. The heated samples were then cooled at −20 °C for 20 min to stop the derivatization reaction and inhibit the decomposition of labeled complexes with analytes, and after dilution with deionized water, they were subjected to separation using a BGE consisting of 20 mM Na_2_B_4_O_7_ and 20 mM SDS at pH 9.0.

The electropherograms obtained from samples analyzed after derivatization with CFSE did not display all the expected peaks corresponding to CFSE-TH derivatives. As a result, the pH of the derivatization mixture was increased to 10.0, which led to the appearance of sharp peaks, albeit with some interference. However, changing the pH of the derivatization mixture to 10.0 led to the appearance of additional sharp peaks in the electrophoretic view, albeit with some interference. In contrast, when using 5-DTAF, clear and strong signals were observed only for analytes with lower molecular weights, such as L-Tyr and DIT. However, compounds with higher molecular weights exhibited relatively weaker signals, making their accurate quantification challenging. The final agent tested was FITC; however, it was observed that during solution preparation in acetone, the presence of water promoted its degradation. Therefore, it was critical to protect the reagent from moisture exposure. Electropherograms of samples analyzed after derivatization with FITC exhibited sharp and intense peaks corresponding to FITC-TH complexes, as well as degradation products, enabling the separation of all analytes. Based on these results and the relatively shorter reaction time compared to the CFSE procedure, it was decided to proceed with optimization using FITC isomer I as the derivatizing agent.

### 2.2. Buffer Optimization

In the preliminary optimization process of the electrophoretic separation method for THs, a universal UV-Vis DAD was employed. Their detection was made possible by the presence of chromophore groups in the structure of THs at the µg mL^−1^ level and after using preconcentration techniques at the concentration of ng mL^−1^. The DAD enabled observation of the entire spectral range (190–300 nm); however, by comparing the peak heights at different wavelengths, it was found that signals were most intense at λ = 200 nm. Consequently, further analyses were conducted at this specific wavelength. The optimization process involved both the qualitative composition of the separation buffer and its pH. The selection of pH was guided by the pKa values of THs, which were approximately 2 for the basic group and around 9 for the acidic group. Initially, acidic buffers were employed, including citrate buffer at pH 3.5, phosphate buffer (25 mM sodium dihydrogen phosphate (NaH_2_PO_4_) with 2% phosphoric acid (H_3_PO_4_)) at pH = 2.0, a 25 mM phosphate buffer with the addition of 10% (*v*/*v*) acetonitrile and 0.5% (*v*/*v*) polyethylene glycol (PEG) 400 at pH = 2.9, and phosphate buffer at pH = 2.25. Only in the case of the last buffer the satisfactory separation of analytes was achieved. Subsequently, the basic pH of the buffers was examined. Na_2_B_4_O_7_ was used as the main component of the BGE in the range of 10–50 mM, with pH adjusted using a 0.1 M sodium hydroxide (NaOH) solution. Na_2_B_4_O_7_-based buffers did not provide complete separation; therefore, it was decided to add SDS as a factor to enhance the separation capabilities of the electrophoretic system. SDS concentrations ranging from 10 mM to 50 mM were tested. The addition of 20 mM SDS to the BGE resulted in satisfactory separation; however, increasing the surfactant concentration to 50 mM caused peak interference in the electrophoretic system. Ultimately, BGE composed of 20 mM Na_2_B_4_O_7_ and 20 mM SDS was chosen as optimal for the separation by MEKC, both with DAD and LIF detection. The basic pH of the BGE proved highly beneficial since FITC (used as derivatizing agent) exhibits a dependence of fluorescence intensity on pH value, with optimal intensity observed at around pH 9.0. It is noteworthy that FITC has two pKa points (8.9 and 10.5), making it a strong anion at pH > 9.0; additionally, both carboxyl and hydroxyl groups of FITC undergo ionization, along with carboxyl groups of THs, resulting in the entire FITC-TH complex being a strong anion. In electrophoretic separation, this is advantageous as anionic compounds migrate towards the anode against the EOF directed towards the cathode. This interaction results in sharp and symmetrical peaks. An attempt was made to investigate the effect of pH on FITC-TH separation, where optimal results were obtained at pH = 9.0.

### 2.3. The Effect of Surfactant Addition to BGE on the Fluorescence Signal Enhancement

**Figure 1:**
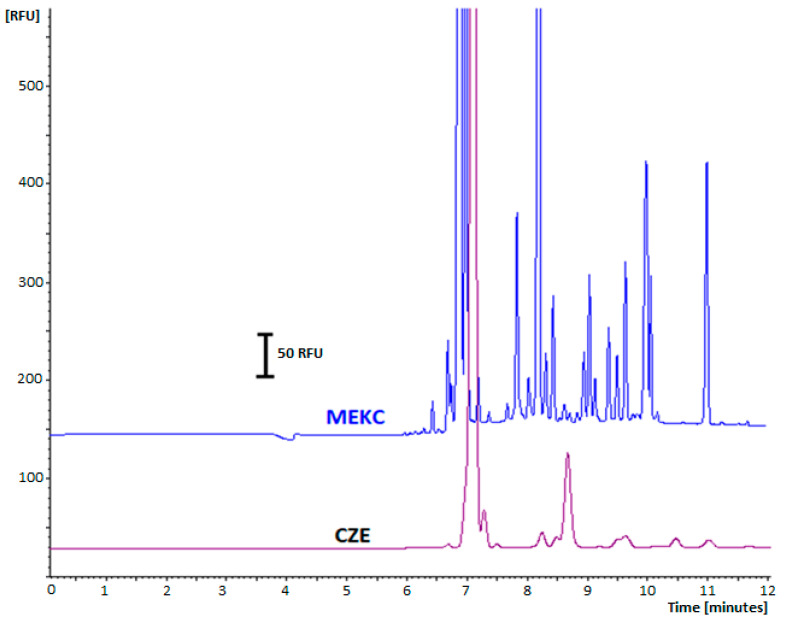
Effect of SDS addition to BGE on the signal amplification and separation efficiency of a urine sample spiked with labeled THs with FITC. LIF detection, 480/520 nm. Electropherograms with PAEKI mode of injection (−5 kV, 60 s, 3.45 kPa): MEKC method with BGE 20 mM Na_2_B_4_O_7_ and 20 mM SDS; CZE method with BGE 20 mM Na_2_B_4_O_7_. Applied voltage 25 kV, capillary 75 μm ID, 60 cm length, analytes concentration: 10 ng mL^−1^ (after dilution with deionized water).

In our study, the fluorescent capabilities of labeled THs in different micellar media such as: anionic (SDS in the range 10–50 mM), cationic cetyltrimethylammonium bromide (CTAB in the range 1–10 mM), and nonionic polyoxyethylene (23) lauryl ether (Brij-35 in the range 0.5–30 mM) surfactants above their critical micelle concentrations were tested. There was a significant enhancement of fluorescence signal intensity in the presence of the anionic surfactant SDS in BGE (MEKC mode) compared to a traditional buffer solution (CZE). It was proven that MEKC employment can improve separation efficiency with UV detection, and it can also enhance the separation of fluorescent analytes, making it a valuable additive in the development of a highly sensitive LIF method for the electrophoretic determination of free THs. The fluorescence enhancement in the presence of SDS was significant when compared with the native fluorescence intensity of the analytes in a buffer without the surfactant (CZE) (Figure 1).

This effect may be attributed to alterations in the physicochemical properties of the solute and the physical properties of the solution [36]. This phenomenon arises from the formation of complexes between the tested compounds and the SDS surfactant, which imparts rigidity to the complexed molecules, thereby restricting their free rotation and reducing the likelihood of non-radiative processes [36,37]. The addition of SDS to the BGE increases its viscosity and likely enhances chemical shielding around the analytes, protecting them from collisions that could lead to energy loss during detection [37,38,39]. Furthermore, the observed increase in fluorescence intensity of FITC-analyte complexes in the presence of the surfactant can be attributed to an enhancement in fluorescence quantum yield. In fluorescence-based techniques, surfactants can be effectively utilized to amplify the fluorescence intensity of target fluorophores, thereby improving both the performance and sensitivity of such techniques for various analytes.

### 2.4. Signal Amplification Using the Modification of Injection Parameters

The typical volume that can be injected into the capillary using the hydrodynamic injection (HDI) method is usually limited to a few percent of the total capillary volume, as larger volumes can lead to band broadening. To mitigate this phenomenon, a sample matrix containing 0.1 mM NaOH was utilized, resulting in stacking effects of the analytes. However, the observed signal enhancement of approximately 5–10 times was insufficient for detecting THs at biological concentrations measured in pg mL⁻^1^. To address this limitation, critical parameters for implementing an online preconcentration technique based on PAEKI were selected, building on optimized parameters from the reference HDI method. PAEKI theoretically allows for unlimited sample injection into the capillary, as signal enhancement in this method is independent of sample volume. In this approach, a sample dissolved in a highly diluted NaOH solution (0.1 mM) was introduced at the capillary inlet while the separation buffer was present at the outlet. Subsequent dilutions of standard concentrations were prepared using a 0.1 mM NaOH solution. Electrokinetic injection (EKI) was performed with reversed electrode polarity: the cathode (negative electrode) was positioned at the inlet, and the anode (positive electrode) was positioned at the outlet. Based on the pKa values of all analytes, it was assumed that free THs would acquire a negative charge under basic conditions (pH 9.3), necessitating EKI with reversed electrode polarity. Additionally, for LIF detection, FITC-TH complexes possessed extra carboxyl groups introduced by isothiocyanate labeling, further enhancing their anionic character under these conditions.

The results obtained with different injection methods confirmed that HDI under pressure enables sample injection without significant deviation in analyte signal values. In contrast, PAEKI showed deviations for charged analytes due to differences in their electrophoretic mobility (Table 3 and Table 4). Furthermore, PAEKI (similar to EKI) favors analytes with high electrophoretic mobility in the same direction as EOF. During PAEKI, sample injection occurs through both EOF, and the analytes’ own electrophoretic movement; therefore, the size and direction of EOF are critical parameters. This mode of injection involves applying a voltage of several kilovolts for a few seconds, with either normal or reversed polarity depending on analyte charge. In this study, reversed polarity was employed during PAEKI for negatively charged THs and their FITC complexes. Specifically, a negative potential was applied to inject TH samples (for UV detection) and their FITC-labeled complexes (for LIF detection) into the capillary, while positive pressure was applied toward the anode (at the outlet vial) to balance EOF.

**Figure 2:**
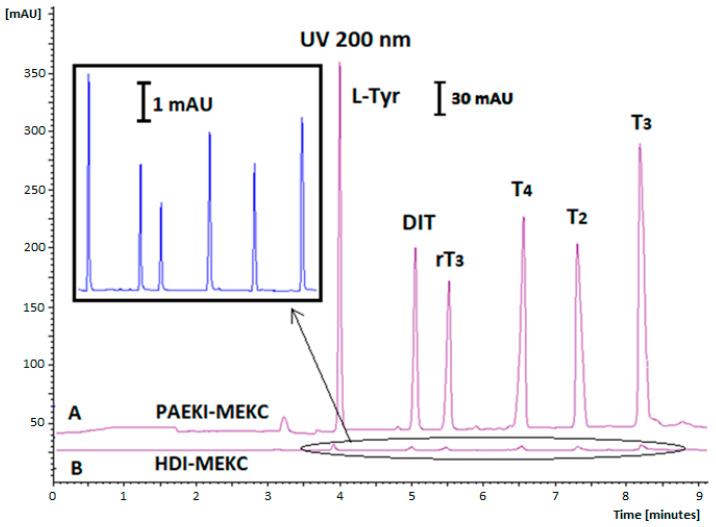
Comparison of PAEKI and typical HDI methods with UV detection (200 nm) for free THs by MEKC, BGE: 20 mM Na_2_B_4_O_7_, and 20 mM SDS; V = 25 kV; (A) PAEKI: −5 kV, 60 s, 3.45 kPa; (B) HDI: 5 s, 3.45 kPa. Analyte concentration: 10 µg mL−1 (without dilution). Other parameters as in Figure 1.

**Figure 3:**
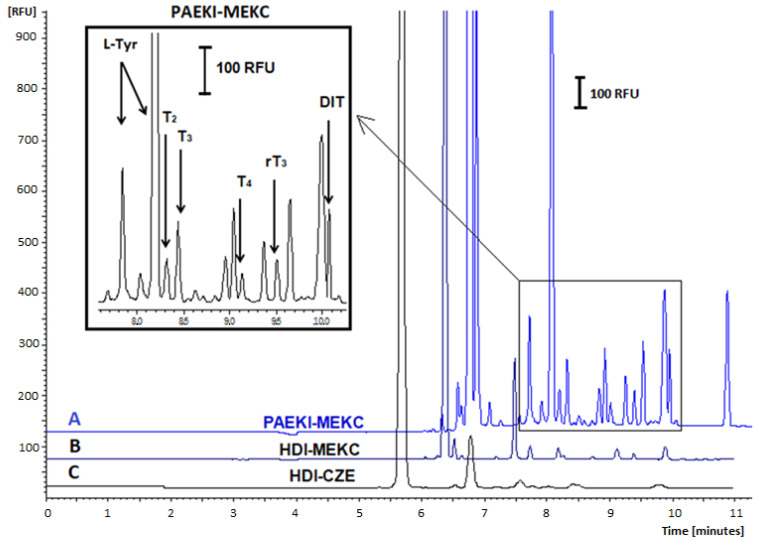
Effect of SDS addition to BGE on the signal amplification and separation efficiency of FITC-labeled THs. LIF detection, 480/520 nm. Electropherograms (A): PAEKI-MEKC method, −5 kV, 60 s, 3.45 kPa, BGE: 20 mM Na_2_B_4_O_7_ and 20 mM SDS; (B) HDI-MEKC method, 5 s, 3.45 kPa, BGE: 20 mM Na_2_B_4f_O_7_ and 20 mM SDS; (C) HDI-CZE method, 5 s, 3.45 kPa, BGE 20 mM Na_2_B_4_O_7_. Analytes concentration: 10 ng mL^−1^ (after dilution). Other parameters as in Figure 1.

Various applied voltage levels for PAEKI were tested, ranging from 1 to 10 kV; using low applied voltage values (1–2 kV) did not yield satisfactory electropherograms due to insufficient conductivity in the sample zone. Conversely, electrokinetic dosing at 8–10 kV resulted in capillary overload with analytes. The duration of dosing (0.5–3 min) and applied pressure levels (0.1–1 psi) were also tested to balance the electrophoretic flow directed towards the cathode (+). The most optimal conditions for the PAEKI method were −5 kV, 0.5 psi (3.45 kPa), and an injection time of 60 s. The effect of THs signal amplification using PAEKI with DAD is shown in Figure 2, while for PAEKI with LIF detection, it is shown in Figure 3.

To evaluate the signal amplification effect for analytes, the enhancement factor (EF) was determined as a ratio of the LODvalue obtained using the PAEKI-MEKC method, with DAD and LIF detection to the LOD parameter calculated after the method based on standard sample dosing (HDI 5 s, 3.45 kPa) with UV detection (Table 3). The LOD and LOQ were calculated as three and ten times the baseline noise level, respectively, for each tested method (data are shown in Table 4).

The EF for the PAEKI-MEKC method with LIF detection was calculated using the traditional HDI-MEKC method with UV detection as a reference. The LOD and LOQ were determined as signal/noise ratio (S/N) of three and ten times, respectively (the data are summarized in Table 3). It can be observed that the LOD values for THs determined by the conventional HDI mode ranged from 45.5 to 113.6 ng mL^−1^, whereas the proposed PAEKI strategy yielded LOD values between 3.6 and 8.9 ng mL^−1^ for UV detection and between 3 and 15.2 pg mL^−1^ (considering dilution). Therefore, the use of labeled THs in combination with the PAEKI technique, employing a longer injection time (60 s) and LIF detection, enabled an increase in sensitivity by EF of between 4685 and 15,149 for the tested compounds compared to the traditional injection (5 s) with UV detection.

### 2.5. Validation Study

In our experiments, using the conventional HDI method with DAD, the concentration of THs ranged from 1 to 20 μg mL^−1^, while for the PAEKI method with the same detector, it ranged from 1 to 1000 ng mL^−1^ (electropherogram Figure 2).

The developed PAEKI-MEKC method with LIF detection was validated using artificial urine samples in accordance with the ICH guidelines for bioanalytical method validation [40]. The linearity of the method was evaluated based on six independent calibration curves, while the accuracy and precision were assessed for the concentration range of 10–10,000 pg mL^−1^ for L-Tyr and 50–10,000 pg mL^−1^ for the other THs (after dilution with deionized water). Artificial urine samples were selected for validation studies due to the presence of free THs in real samples. A volume of 0.5 mL of urine was used for calibration and quality control (QC) samples. Urine samples were spiked with all analytes at concentrations of 50, 100, 500, 1000, 5000, and 10,000 ng mL^−1^ and an additional concentration of 10 ng mL^−1^ for L-Tyr. QC urine samples were spiked with all analytes at concentrations of 100, 1000, and 10,000 ng mL^−1^ to generate low (LQC), medium (MQC), and high (HQC) QC samples, respectively, and diluted with deionized water before analysis (10,000-fold for L-Tyr and 1000-fold for other analytes).

The samples were subsequently deproteinized and subjected to protein ballast removal, followed by an SPE procedure, as described in Section 2.4. Quantitative analysis of derivatized samples was performed using the elaborated method after appropriate dilution with deionized water prior to injection. The developed method demonstrated reliable linear responses across the validated concentration ranges for each analyte.

For PAEKI with LIF, the LOD for free THs (L-Tyr, T2, T3, rT3, T4, and DIT) ranged from 3 to 15.2 pg mL^−1^ (including sample dilutions during the derivatization procedure and a 10,000-fold dilution prior to separation for L-Tyr and a 1000-fold dilution for the other analytes). The LOQ ranged from 9.9 to 49.8 pg mL^−1^ (also including these dilutions) (Table 4). For each analyte, calibration curves after FITC derivatization exhibited linearity with high coefficients of determination (r^2^ > 0.998).

Precision was expressed as relative standard deviation (% RSD), while accuracy was calculated as the deviation of the mean from the nominal concentration for THs. As presented in Table 5, RSD values for intra-day precision (*n* = 6) ranged from 3.2% to 11.0%, while inter-day precision (*n* = 6) ranged from 4.1% to 12.7%. Accuracy was confirmed by spiking urine samples with three concentrations of standard analytes, applying derivatization, and calculating recovery ratios. The accuracies ranged from 97.2 to 103.8% for intra-day variability and from 96.7 to 104.9% for inter-day variability.

The stability of the formed complexes was evaluated at 4 °C. All FITC-labeled analytes remained stable for up to 12 h; however, after 24 h, the peak intensity of the derivatized FITC-TH complexes decreased below 90%, confirming the lack of long-term stability of these derivatives.

**Figure 4:**
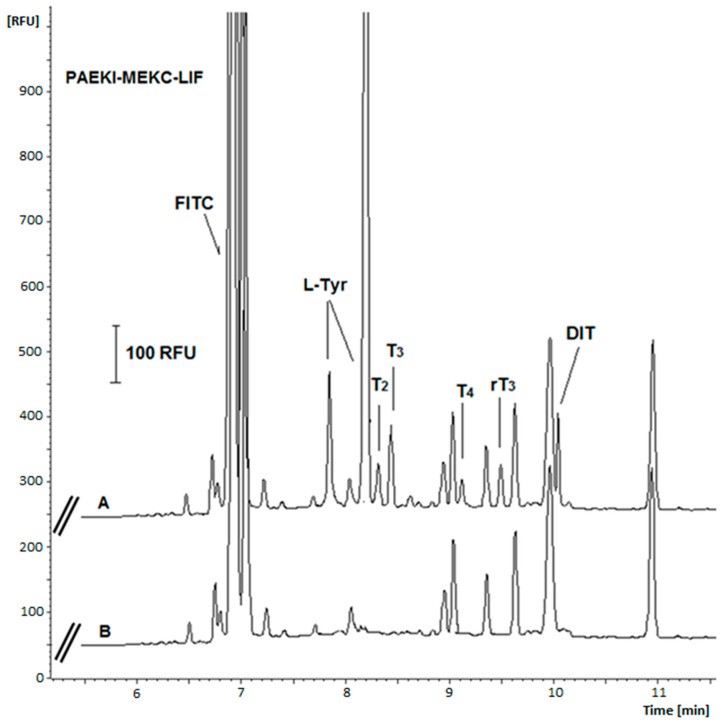
Comparison of electropherograms of FITC-derivative urine sample spiked with analytes (A) and derivatized artificial urine sample (blank sample) (B). Analytes concentration: 10 ng mL^−1^. Samples were diluted 1000 times with deionized water before analysis. Other parameters as in Figure 1.

The selectivity of the method was verified by applying it to blank and spiked artificial urine samples for peak co-migration. The results of these tests indicated that the urine sample ballast did not interfere with the signals of any analyte (Figure 4). Similarly, the electrophoretic repeatability of the developed method was determined as the RSD of peak height and migration times calculated for six replicate PAEKI analyses of the standard sample (at 1000 pg mL^−1^ for each drug). Despite the use of PAEKI, which is characterized by lower repeatability of results than HDI mode, values lower than 10.1% for peak heights and lower than 3% for migration times were obtained (Table 4).

In all tested electrophoretic conditions, the selectivity and efficiency of the developed electrophoretic method remained unchanged, which also proves the robustness of the proposed method. The precision and accuracy results showed acceptable values for both intra- and inter-day assays for all the analytes. These results confirm the suitability of the proposed method for the determination of free THs in the tested urine samples.

## 3. Materials and Methods

### 3.1. Chemicals and Reagents

All the chemicals used in the experiment were of analytical-reagent grade. Standard solutions containing 1 mg mL^−1^ of each analyte were prepared using THs, including L-TYR, T_2_, T_3_, T_4_, rT_3_, and DIT, obtained from Sigma-Aldrich (St. Louis, MO, USA). These standard solutions were stored at −18 °C in a frozen state, and further dilutions were made as required using Milli-Q water. For the derivatization process, CFSE, 5-DTAF, and FITC were purchased from Sigma-Aldrich (St. Louis, MO, USA). Other organic solvents of chromatographic-grade purity, SDS, Brij-35, and CTAB were obtained from Sigma-Aldrich (St. Louis, MO, USA). The background electrolyte (BGE) and derivatization buffer were prepared using Na_2_B_4_O_7_, (Na_2_HPO_4_) or Na_2_CO_3_, and sodium hydroxide (NaOH) for pH adjustment, all sourced from POCh (Gliwice, Poland). Artificial urine (SAE0074-50ML) was purchased from Sigma-Aldrich (St. Louis, MO, USA). Deionized water with a resistance higher than 18 MΩ was obtained using a Millipore Milli-Q purification system (Bedford, MA, USA).

### 3.2. CE Instrumentation

A Beckman P/ACE MDQ system (Beckman Coulter, Fullerton, CA, USA) equipped with a LIF detector (excitation: 488 nm; emission: 520 nm) or with DAD and a liquid cooling device was employed. All analytes were analyzed using an uncoated fused-silica capillary (Polymicro Technologies, Phoenix, AZ, USA) measuring 50.2 cm in total length (effective length: 40 cm) and with an inner diameter of 75 µm. The temperature of the separation was maintained at 25 ± 0.1 °C by immersing the capillary in a cooling liquid circulating within the cartridge tube. The new capillary was conditioned with 0.1 M NaOH for 10 min, followed by deionized water for 2 min. Routine washing between runs was performed daily using pressure with 0.1 M NaOH (1 min), deionized water (1 min), and rinse buffer (1 min) under positive pressure (20 psi) applied at the injection end. A 32 Karat System Software Version 5.0 P/N 715080 (Beckman Coulter) was used for data processing.

### 3.3. Derivatization Procedure

For the derivatization procedure, a 10 μL volume of the standard solutions of all analyzed THs was mixed with 10 μL of a 5 mM FITC solution in dimethyl sulfoxide (DMSO) to achieve the appropriate ratio of the total concentration of analytes to FITC. The mixture was then brought to a final volume of 0.1 mL with a 0.2 M Na_2_HPO_4_ solution adjusted to pH 9.5. In an Eppendorf tube, the mixture was vortexed and allowed to react in the dark at 60 °C for 20 min. Subsequently, the samples were immediately placed in a refrigerator at 4 °C to slow down the decomposition reaction of the labeling agent. Electrophoretic analyses confirmed that this approach yielded an electropherogram with the fewest peaks of FITC decomposition products, indicating its effectiveness. Following derivatization, the samples were diluted 1000-fold with deionized water before being injected into the MEKC-LIF system. For L-TYR, it was necessary to dilute the sample 10,000 times due to its strong signal intensity. A blank solution (without analytes) was prepared simultaneously for each determination. The reaction of L-TYR derivatization with FITC involves a nucleophilic attack by the derivatizing reagent on the amino group of the amino acid—specifically, tyrosine. The alkaline environment is crucial for this reaction; at such a pH, FITC exhibits the most intense fluorescence, and the amino group of tyrosine remains uncharged. Our studies have demonstrated the reproducibility of results for samples prepared within one day (up to 12 h from the start of the derivatization reaction) while simultaneously confirming compliance with validation requirements. A key factor in achieving reproducible results was the immediate cooling of the sample containing labeled analytes upon completion of the derivatization reaction and storing it at 4 °C until analysis.

### 3.4. Extraction Procedure

Due to the presence of free THs in natural urine samples, artificial urine samples were used for validation studies. Furthermore, because of the relatively high content of various ballast compounds in biological matrices, it was necessary to deproteinize the samples beforehand. For this purpose, 0.5 mL of acetone was added to 0.5 mL of the samples, which were then vortexed and centrifuged at 14,000 rpm. Next, 50 mM ammonium acetate (at pH 7.0) was added to the acetone-water solutions containing analytes to avoid sample acidification. This step prevents co-elution of polar uric acids during the elution procedure and may lead to a purer extract that was subjected to a further solid-phase extraction procedure using EVOLUTE^®^ EXPRESS AX columns that are based on a modified non-polar polystyrene-divinylbenzene polymer, which incorporates polar hydroxyl groups. These non-ionizable hydroxyl groups can ensure that the polymer is both highly water wettable and also able to extract a diverse range of analytes through non-polar (van der Waals) interactions. The eluent (0.5 mL of methanol) was evaporated to dryness at 50 °C under vacuum (Labconco, Kansas City, MO, USA) for 1 h. The next step was to dissolve the residue in a solution of 0.1 mM NaOH. After reconstituting the solution with analytes, the derivatization reaction using FITC was carried out according to the optimized procedure described in the Section 2.

## 4. Conclusions

This article discusses and compares various approaches for the determination of THs using electromigration techniques, including DAD and LIF detection. For the first time, water-wettable EVOLUTE EXPRESS AX SPE columns were applied for the extraction of THs from urine samples. Moreover, three derivatization agents—5-DTAF, CFSE, and FITC isomer I—were compared. The most promising results were obtained using FITC. It is possible that steric hindrance associated with the derivatives of THs influenced the derivatization process, as the FITC molecule has the smallest reactive isothiocyanate group.

A particularly intriguing aspect of this study was the use of SDS micelles in the BGE and proving their impact on signal enhancement. While the exact mechanism remains unclear, it is hypothesized that the formation of SDS-FITC-TH complexes, due to their high molecular weight, restricts the free rotation of these macromolecules. This restriction likely reduces energy loss from excitation states, thereby significantly enhancing signal intensity. The influence of micellar complexes on signal amplification using LIF detection will be further investigated by our research team in future studies.

Another novelty was the use of the online preconcentration technique—PAEKI—to increase the sensitivity of FITC-TH complexes. Moreover, due to the anionic nature of both TH and FITC-labeled TH, the PAEKI technique was used to determine these compounds for UV and LIF detection for the first time. This approach achieved LOD for L-Tyr, T2, T3, rT3, T4, and DIT ranging from 3 to 15.2 pg mL^−1^. Compared to conventional HDI methods, a remarkable 15,149-fold signal amplification was observed. Given the broad scope of this research and the ambitious objectives set forth, further results, including analyses performed on samples from healthy volunteers, will be presented elsewhere.

The developed MEKC-PAEKI-LIF procedure is selective, reproducible, and efficient, as demonstrated by the validation parameters presented in Section 2.5. This study can serve as a reliable foundation for future determinations of THs using electromigration techniques in various samples, such as pharmaceuticals and biological matrices.

## Figures and Tables

**Table 1:** Separation methods for the determination of THs.

Analytes	Separation Technique	LOD	Extraction Technique	Sample Matrix	Reference
T4, T3, rT3, T2, rT2, T1	isotope dilution LC-MS/MS	0.6–1.1 ng mL^−1^	SPE	Rat brain and thyroid gland	[1]
T4, T3, rT3, T2, rT2	LC-MS/MS	0.3–3.9 ng L^−1^	SPE	Waste water	[2]
T4, T3, rT3, T2	LC-ESI-MS/MS	0.34–1.4 ng mL^−1^	SPE	Serum	[25]
T4, T3	LC-MS	1.0 ng L^−1^	LLE, SPE	Plasma	[26]
T4, T3	HPLC-UV	1–2 ng	SPE	Urine, plasma	[27]
T4, T3, T2, L-Tyr, DIT, MIT	HPLC-UV	0.02–0.1 ng µL^−1^	SPE	Urine, plasma	[28]
T4	UHPLC-MS/MS	0.64–0.79 ng L^−1^	SPE	Waste water	[29]
T4, T3, rT3, T2, rT2	UPLC-MS/MS	0.16–0.59 pg	LLE, SPE	Human and animal tissues	[30]
T4	GC/LC-MS	0.16 µM	LLE, SPE	Plasma	[31]
T4, T3, rT3, T2, MIT, DIT	CE-UV	0.54–1.43 µg L^−1^	IP-HF-LLLME	Serum	[32]
T4, T3	CE-AD	0.085–0.1 µM	-	Pharmaceuticals	[33]
T4, T3, Iodides	CE-ICP-MS	0.08–3.5 µg L^−1^	-	Serum, urine	[34]
L-Tyr, T2, T3, rT3, T4, DIT	PAEKI-LIF	* 3.0–15.2 pg mL^−1^	SPE	Urine	This study

MIT—3-monoiodotyrosine, LLE—liquid-liquid extraction, IP-HF—LLLME–ion pair hollow fiber liquid-liquid-liquid microextraction; * values after dilution with deionized water.

**Table 2:** Parameters of the labeling reaction for THs with selected derivatization agents under optimum experimental conditions.

Derivatization Agents	Parameter of Derivatization Procedure							
	Derivatization Agent			Buffer Solution (pH 9.5)		Analytes Volume (µL)	Time (h)	Temp. (°C)
	Solvent	Concentration (mM)	Volume (µL)	Concentration and Type of Buffer	Volume (µL)			
5-DTAF	DMSO	5	5	0.5 M Na_2_CO_3_	20	75	1	40
CFSE	Acetone	5	5	0.1 M Na_2_B_4_O_7_	20	75	24	20
FITC		1.5	10	0.2 M Na_2_HPO_4_	40	50	2	40
Dilution prior the electrophoretic separation 1000 times (or 10,000 times for L-Thy) with deionized water for all samples. Na_2_CO_3_-sodium carbonate, Na_2_HPO_4_-disodium hydrogen phosphate								

**Table 3:** Comparison of LOD values for free THs and determined EFs for the PAEKI technique with UV and LIF detection versus traditional HDI.

Analyte	LOD				EF	
	UV [ng mL^−1^]		LIF [pg mL^−1^]		UV PAEKI/HDI	LIF-PAEKI/ UV-HDI
	HDI	PAEKI	HDI *	PAEKI *		
L-Tyr	45.5	3.6	80.5	3.0	26.8	15,149
T2	78.1	7.2	306.1	14.7	23.3	7467
T3	56.8	4.8	116.2	8.5	29.6	4685
rT3	113.6	8.9	355.2	15.2	20.9	5322
T4	62.5	6.3	394.6	13.3	13.7	6702
DIT	78.1	7.5	117.8	7.6	15.5	10,300

* Dilution of samples with deionized water prior the electrophoretic separation 10,000-fold for L-Tyr and 1000-fold for other analytes.

**Table 4:** Validation parameters for PAEKI-MEKC-LIF method and precision data for migration times and peak signal for HDI-UV and PAEKI-MEKC-LIF.

Analyte	PAEKI-MEKC-LIF							HDI-UV
	Slope	Intercept	*R* ^2^	LOD [pg mL^−1^]	LOQ [pg mL^−1^]	Precision (%RSD)		
						Migration Time	Peak Hight Signal	Peak Hight Signal
L-Tyr *	2090.0	−836.8	0.9997	3.0	9.9	2.1	7.7	4.1
T_2_	512.5	−1811.2	0.9985	14.7	48.4	2.4	8.4	4.8
T_3_	1057.4	−4785.4	0.9991	8.5	28	2.5	9.4	5.3
rT_3_	595.5	−9599.2	0.9984	15.2	49.8	2.7	10.1	5.8
T4	396.1	−17,551	0.9989	13.3	44	2.8	9.2	5.1
DIT	2025.8	10,167	0.9990	7.6	25	2.4	8.2	4.3

* L-Tyr diluted 10,000-fold with deionized water before analysis.

**Table 5:** The precision and accuracy data obtained for PAEKI-MEKC-LIF method for quality control samples: LQC (100 pg mL^−1^, MQC (1000 pg mL^−1^) and HQC (10,000 pg mL^−1^).

Analyte	Nominal Concentration [pg mL^−1^]	Intra-Day (*n* = 6)			Inter-Day (*n* = 6)		
		Observed Concentration [pg mL^−1^] (mean ± SD)	Accuracy [%]	Precision [CV%]	Observed Concentration [pg mL^−1^] (mean ± SD)	Accuracy [%]	Precision [CV%]
L-Tyr	LQC	98.4 ± 3.2	98.4	8.4	97.8 ± 4.4	97.8	9.5
	MQC	1003.2 ± 19.2	100.3	5.3	1004.5 ± 25.4	100.4	7.7
	HQC	9993.1 ± 87.5	99.3	3.2	9991.7 ± 89.8	99.9	4.4
T2	LQC	102.2 ± 5.2	102.2	11.0	104.9 ± 6.9	104.9	12.7
	MQC	1004.9 ± 19.8	100.5	7.9	1008.1 ± 22.4	100.8	8.3
	HQC	9988.6 ± 84.3	99.9	5.1	9985.6 ± 98.3	99.9	4.5
T3	LQC	97.2 ± 5.2	97.2	9.8	97.0 ± 5.9	97.0	10.7
	MQC	995.0 ± 24.4	99.5	8.9	993.3 ± 28.3	99.3	9.2
	HQC	9989.7 ± 89.7	99.9	4.4	9978.0 ± 90.2	99.8	7.5
rT3	LQC	103.8 ± 7.2	103.8	9.9	104.2 ± 8.8	104.2	11.8
	MQC	1005.8 ± 26.7	100.6	8.0	1007.8 ± 29.3	100.8	8.7
	HQC	9982.0 ± 83.2	99.8	4.3	9964.0 ± 91.6	99.6	6.2
T4	LQC	103.7 ± 5.6	103.7	9.1	104.5 ± 7.6	104.5	10.4
	MQC	985.9 ± 30.6	98.6	6.5	983.4 ± 32.7	98.3	7.8
	HQC	10,021.6 ± 91.6	100.2	3.2	10,044.6 ± 97.0	100.5	4.1
DIT	LQC	97.9 ± 3.2	97.9	9.0	96.7 ± 5.2	96.7	9.8
	MQC	997.9 ± 17.6	99.8	7.2	995.8 ± 21.9	99.6	8.4
	HQC	10,037.5 ± 68.3	100.4	4.0	10,049.3 ± 75.3	100.5	6.0

## Data Availability

Data are contained within the article.
